# Oleanolic acid alleviates obesity‐induced skeletal muscle atrophy via the PI3K/Akt signaling pathway

**DOI:** 10.1002/2211-5463.13780

**Published:** 2024-02-17

**Authors:** Yaqin Sun, Xiaofang Wei, Tong Zhao, Hongwei Shi, Xiaojing Hao, Yue Wang, Huiling Zhang, Zhichao Yao, Minxing Zheng, Tianyun Ma, Tingting Fu, Jiayin Lu, Xiaomao Luo, Yi Yan, Haidong Wang

**Affiliations:** ^1^ College of Veterinary Medicine Shanxi Agricultural University Jinzhong China

**Keywords:** dexamethasone, muscle atrophy, obesity, oleanolic acid, PI3K/Akt signaling pathways

## Abstract

Oleanolic acid (OA) is a pentacyclic triterpene with reported protective effects against various diseases, including diabetes, hepatitis, and different cancers. However, the effects of OA on obesity‐induced muscle atrophy remain largely unknown. This study investigated the effects of OA on skeletal muscle production and proliferation of C2C12 cells. We report that OA significantly increased skeletal muscle mass and improved glucose intolerance and insulin resistance. OA inhibited dexamethasone (Dex)‐induced muscle atrophy in C2C12 myoblasts by regulating the PI3K/Akt signaling pathway. In addition, it also inhibited expression of *MuRF1* and *Atrogin1* genes in skeletal muscle of obese mice suffering from muscle atrophy, and increased the activation of PI3K and Akt, thereby promoting protein synthesis, and eventually alleviating muscle atrophy. Taken together, these findings suggest OA may have potential for the prevention and treatment of muscle atrophy.

## Graphical abstract

Muscle atrophy occurs in certain muscles with inactivity in many diseases, and there is a lack of effective therapies at present. We report here that oleanolic acid (OA) inhibits expression of *MuRF1* and *Atrogin1* genes in skeletal muscle of obese mice suffering from muscle atrophy and increases protein synthesis through the PI3K/Akt signaling pathway, thereby alleviating muscle atrophy.

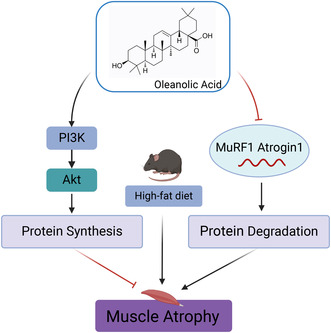

**Abbreviations**
Atrogin1: muscle atrophy F‐box
BAT: brown adipose tissue
CCK‐8: Cell Counting Kit‐8
CSA: cross‐sectional area
Dex: dexamethasone
DMEM: Dulbecco's modified Eagle's medium
GAS: gastrocnemius
HFD: high‐fat diet
IGF‐1: insulin‐like growth factor 1
iWAT: inguinal white adipose tissue
MuRF1: muscle ring finger 1
OA: oleanolic acid
PVDF: polyvinylidene fluoride
pWAT: perirenal white adipose tissue
SDS/PAGE: SDS‐polyacrylamide gel electrophoresis
TA: tibialis anterior
WAT: white adipose tissue

Skeletal muscle is widely distributed in the human body, and it is the largest organ system. Skeletal muscle not only directly participates in sports, but also acts as an important secretory organ to secrete muscle factors affecting the function of other organs. In addition, skeletal muscle is often affected by various factors such as exercise and disease [1]. Therefore, the maintenance of skeletal muscle function is of great significance to body health [2, 3]. Muscular dystrophy is defined as a decline in muscle mass and functions. Many conditions can lead to muscle atrophy such as obesity, limb disuse, heart failure, cachexia, and Duchenne muscular dystrophy [4, 5].

Obesity is associated with both loss of skeletal muscle mass and dysfunction, and it is also known as sarcopenic obesity [6, 7]. Obesity induced by high‐fat diet (HFD) can reprogram the expression of skeletal muscle factors and increase the expression of muscle ring finger 1 (*MuRF1*) and muscle atrophy F‐box (*Atrogin1*) [8, 9, 10, 11]. *MuRF1* and *Atrogin1* are the main markers of muscle atrophy. The obesity‐induced muscle atrophy is characterized by the decreased muscle mass and cross‐sectional area (CSA). Secondly, the expression of muscle atrophy‐related factors is increased in obese mice, while the expression of muscle generation‐related factors is decreased [12]. Inflammatory cytokines, myostatin‐related signaling pathways, and protein synthesis and degradation pathways are involved in the regulation of muscle atrophy [13] Although many studies have been conducted to explore the drugs to treat muscle atrophy, no effective drug is available to cure it [2].

Protein synthesis and degradation processes are complementary and interrelated, and they play an important role in determining skeletal muscle mass and muscle fiber size [5]. When the rate of protein degradation exceeds that of protein synthesis, muscle atrophy occurs, thus leading to the loss of body weight and muscle mass and the increase in disease incidence [5, 14]. Therefore, it is essential to explore a method or drug to promote muscle growth and development mainly by increasing protein synthesis and/or by decreasing protein degradation. Recent studies have identified some pathways that regulate skeletal muscle protein balance and induce muscle regeneration after injury, which is conducive to the treatment of muscle atrophy [15, 16].

Oleanolic acid (OA) is a natural triterpene compound, and it is not only distributed in many herbs [17], but also in a wide range of fruits [10, 18]. OA has multiple functions including resistance to viruses [18], tumors, and diabetes [19] and protection of liver function [20]. Recent evidence suggests that OA plays an important protective role in obesity and other metabolic diseases by regulating the expression of pro‐inflammatory cytokines [21, 22, 23, 24]. At the same time, OA can resist the adverse effects of obesity by improving adipose mass and impaired glucose tolerance in obese mice [25]. In addition, obesity potentiates glucocorticoid‐induced muscle atrophy [26]. Therefore, we speculate whether OA would improve muscle atrophy caused by obesity. Protein synthesis and degradation processes are complementary and interrelated, and they play an important role in the development and atrophy of skeletal muscle. Ultimately, we speculated that OA would stimulate protein synthesis.

Although numerous studies have confirmed a variety of biological activities of OA, the effects of OA on obesity‐induced muscle atrophy have rarely been reported. In this study, we examined the effects of OA on skeletal muscle mass and the CSA of muscle fiber in obese mice. We found that OA improved obesity‐induced skeletal muscle atrophy. Furthermore, we sought to decipher the underlying molecular mechanism by investigating HFD‐induced muscle atrophy in obese mice and dexamethasone (Dex)‐induced muscle atrophy in C2C12 myoblasts.

## Materials and methods

### Experimental animals

Male C57BL/6 mice (5 weeks old) were raised at 21–25 °C, 50–60% humidity, and a 12 h light/12 h dark cycle with free access to food and water. Mice were fed with standard rodent chow and water. C57BL/6 male mice were randomly divided into three groups, namely, CHOW group (control), High‐fat diet (HFD) group, and HFD + OA group. HFD and HFD + OA groups were fed with a high‐fat diet with 60% calories derived from fat (XTHF60, Jiangsu Xietong Pharmaceutical Bio‐engineering Co, Ltd.) [27, 28, 29]. After 2 weeks of adaptation, HFD + OA group was gavaged with OA (50 mg·kg^−1^, HY‐N0156, MCE) once a week for a total of 6 months [8, 30].

At the end of the experiment, blood was sampled from the mouse eyeballs. After sampling, blood samples were stood at room temperature for 30 min and centrifuged at 2000–3000 ***g*** for 10 min. Adipose and muscle tissues were collected for follow‐up experiments.

### Ethical approval

The study has been approved by the Institutional Animal Care and Use Committee of Shanxi Agricultural University (SXAU‐EAW‐2022M.MJ.008012001). All methods were carried out in accordance with relevant guidelines and regulations.

### Cell cultures

The mouse C2C12 myoblasts were purchased from Kunming Cell Bank, Chinese Academy of Sciences. C2C12 myoblasts were cultured in Dulbecco's modified Eagle's medium (DMEM) supplemented with 10% fetal bovine serum, 100 U·mL^−1^ penicillin, and 100 μg·mL^−1^ streptomycin at 37 °C in a humidified atmosphere (5% CO_2_). The myoblasts were seeded in a 6‐well plate (1 × 10^5^ cells per well) or 96‐well plate (0.2 × 10^4^ cells per well) according to experiment objectives.

### Cell drug treatments

When the cell confluence reached 70–80%, C2C12 cells were treated with the drug and divided into four groups: Control group (without drug additions), NC group (anhydrous ethanol and dimethyl sulfoxide), Dex group (48 h treatment with 50 μm Dex), and Dex + OA group (12 h treatment with 50 μm DEX, followed by 36 h co‐treatment with 50 μm Dex and 60 μm OA). Dex solution (with anhydrous ethanol as the carrier) and OA solution (with dimethyl sulfoxide as the carrier) were configured as stock concentrations of 12.5 and 10 mm, respectively according to the instructions. Then, they were diluted with a suitable medium to the desired concentration. In order to control variables, the proportion of anhydrous ethanol and dimethyl sulfoxide in NC group, Dex group, and Dex + OA group was completely consistent. C2C12 cells were treated with Dex for 12 h and then with Dex and OA for 36 h.

### Cell proliferation assay

First, 100 μL cell suspension containing 2000 cells was added to a 96‐well plate. After 24 h pre‐culture, cells were divided into different groups and treated with drug solutions. After treatment, cell solution was added with 10 μL Cell Counting Kit‐8 (CCK‐8) solution and incubated for 2 h. Absorbance at 450 nm was measured with an enzyme label. According to cell number, the proliferation of cells was evaluated.

### EdU staining

Firstly, 100 μL cell suspension was added into 96‐well plates, pre‐cultured for 24 h, and treated with drug solutions for 48 h. After pre‐culture, the media were discarded. Then, pre‐cultured cells were added with 100 μL EdU working solution and incubated for 2 h for EdU staining. After incubation, work solution then discarded. The incubated cells were fixed with 4% paraformaldehyde (50 μL) at room temperature for 15 min. The cells were washed with 100 μL washing solution (1× PBS) three times for 3 min each time. The cells were incubated in 50 μL permeable solution (PBS) containing 0.3%Triton X‐100 at room temperature for 15 min. The cells were washed with PBS three times for 3 min each time. The each well containing treated cells was added with 50 μL click additive solution (consisting of 43 μL click reaction buffer, 2 μL CuSO4, 0.1 μL Azide 594, and 5 μL click additive solution), and 96‐well plate was shaken gently to make click additive solution fully cover the cells. The cells were incubated at room temperature in the dark for 30 min. The click reaction solution was discarded, and the cells were washed with PBS three times for 3 min each time. After washing, 1× Hoechst was added into the plate well with 50 μL per well, and the cells were incubated for 10 min at room temperature in the dark. 1× Hoechst was discarded, and the cells were washed with PBS three times for 3 min each time. Finally, the cells were observed with a fluorescence microscope, and cell images were captured.

### mRNA extraction and real‐time quantitative PCR

Total RNA including low‐molecular‐weight RNA was extracted from frozen tissues and cell lines using The RNAiso Plus (Takara, Tokyo, Japan) according to the manufacturer's protocol. We used SYBR‐based qRT‐PCR (Monad, Suzhou, China) to quantify the mRNA expression of *MuRF1, Atrogin1, MyoD* and *MyoG*. The relative expression levels were calculated using the comparative‐Ct method ($2^{-\Delta \Delta C_{T}}$ method). The primer sequences are listed in Table S1.

### Western blotting

Cells were lysed in a radio immunoprecipitation assay buffer (RIPA buffer) (P0013B, Beyotime, Shanghai, China) to obtain cell lysate. The concentration of total protein was determined by a BCA™ protein assay kit (P0011, Beyotime). Cell lysate containing 20 μg protein was subjected to a 10% gradient SDS‐polyacrylamide gel electrophoresis (SDS/PAGE) to obtain target protein. The resultant protein was transferred to a polyvinylidene fluoride (PVDF, 0.45 μm) membranes (Millipore, Bedford, MA, USA) and blocked with 5% skim milk. The membranes containing protein were incubated with the primary antibodies against GAPDH (10494‐1‐AP, Proteintech, Wuhan, China), MuRF1 (55456‐1‐AP, Proteintech), Atrogin1 (A3193, ABclonal, Wuhan, China), MyoD (TA7733, Abmart, Shanghai, China), MyoG (sc‐12732, SANTA CRUZ, Dallas, TX, USA), Akt (40 569, SAB, Greenbelt, MD, USA), p‐Akt (4060S, Cell Signaling, Danvers, MA, USA), PI3K (60225‐1‐lg, Proteintech), and p‐PI3K (AF3242, Affinity, Jiangsu, China) at 4 °C overnight. Then, the membranes were washed three times with TBST buffer containing 1× Tris‐buffered saline (pH 7.4) and 0.1% Tween‐20 for 15 min. The membranes were incubated with the secondary antibody (anti‐mouse IgG or anti‐rabbit IgG) for 1 h at 37 °C and washed three times with 1× TBST buffer. The protein signals were measured under a ChemiDoc™ XRS+ Imaging System (Bio‐Rad, Hercules, CA, USA), and the protein band density was quantified by densitometry using the imagej programming software (imagej, NIH, Bethesda, MD, USA). The expression of each target protein was normalized with GAPDH as an internal control.

### Histology of skeletal muscle

Fresh tibialis anterior (TA) muscle was fixed in 4% paraformaldehyde, embedded in paraffin, and 6 μm sections were prepared. The TA sections were stained with hematoxylin and eosin. The images were captured with an inverted microscope (Nikon eclipse Ts2R), and the average cross‐sectional area (CSA) was measured by microscopic analysis. Masson's trichrome staining was performed using Masson's Trichrome Stain Kit following the manufacturer's instructions (G1006, Servicebio). TA muscle fibrosis was quantified by using image j. Collagen volume fraction was calculated as the ratio of the collagen‐positive blue area to the total tissue area.

### Glucose tolerance and insulin resistance tests

To test glucose tolerance, mice were fasted for 15 h, and then intraperitoneally injected with 2 g·kg^−1^ glucose. At 0, 30, 60, 90, and 120 min after glucose injection, blood glucose levels were measured using a blood glucose meter at the mouse tail notch.

To test insulin resistance, mice were fasted for 6 h, and then intraperitoneally injected with 0.75 IU·kg^−1^ recombinant human insulin. At 0, 30, 60, 90, and 120 min after insulin injection, blood glucose concentrations were determined.

### Statistical analysis

Data were expressed as LSM ± SE. Statistical analyses were performed using graphpad prism, version 8.3.1 (San Diego, CA, USA). An unpaired *t*‐test was used to assess differences between two groups.

## Results

### HFD results in the increase in body weight and white adipose tissue mass and loss of muscle mass in mice

**Fig. 1:**
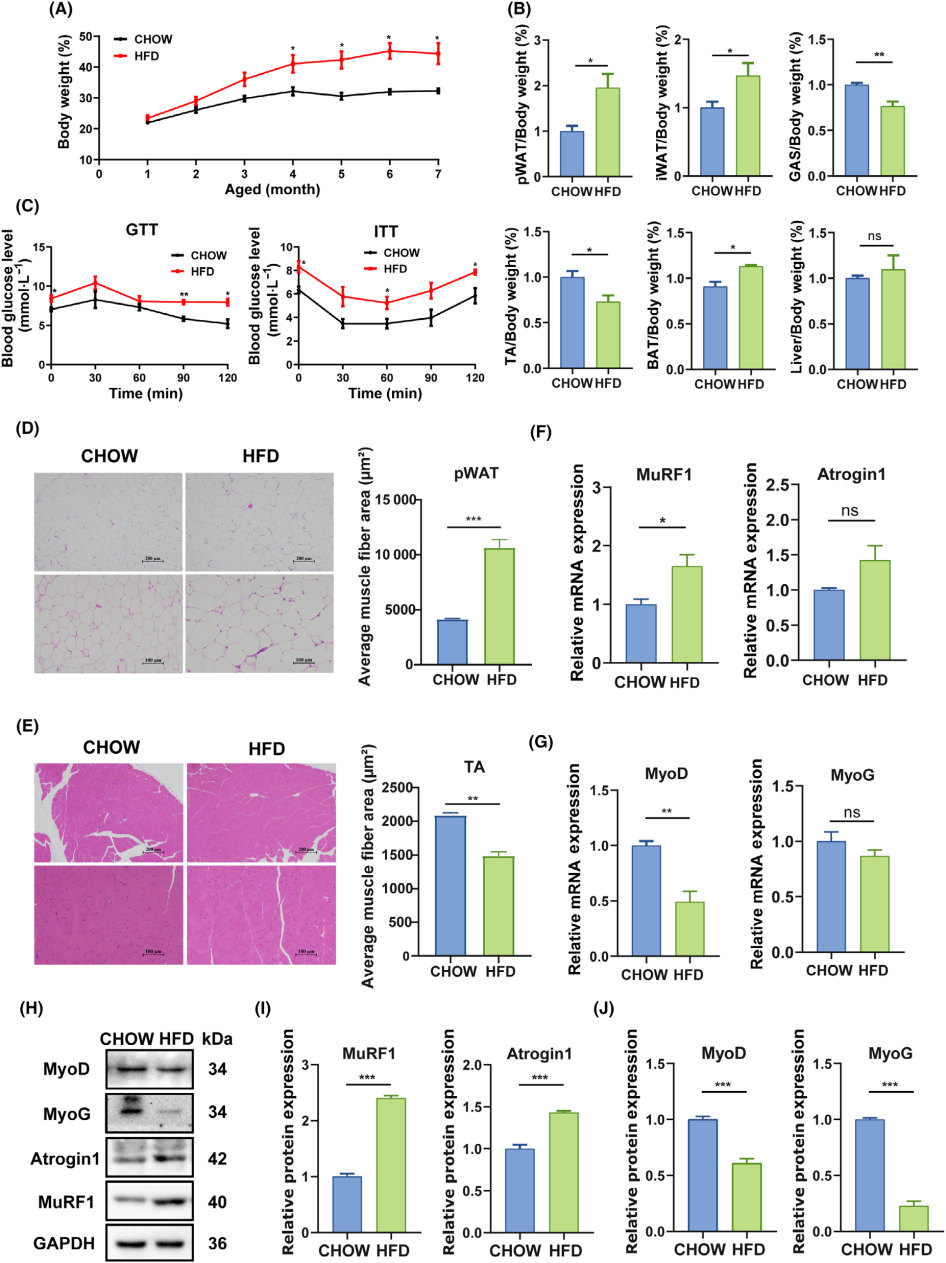
Effects of obesity on body weight and tissue mass in mice. One‐month C57BL/6 mice were fed with high‐fat diet (HFD) for 6 months. (A) Comparison of body weight between CHOW mice and HFD mice (*n* = 4 per group). (B) The ratio of adipose, muscle, liver, or brown adipose tissue (BAT) weight to body weight in CHOW mice and HFD mice (*n* = 4 per group). (C) Glucose tolerance and insulin resistance in CHOW mice and HFD mice (*n* = 4 per group). (D) Hematoxylin and eosin (HE) staining of perirenal white adipose tissue (pWAT). Comparison of average cross‐sectional area of pWAT between CHOW group and HFD group (*n* = 3 per group). Scale bar, 200 μm, 100 μm. (E) HE staining of tibialis anterior (TA) muscle tissue. Comparison of average cross‐sectional area of TA tissues between CHOW group and HFD group (*n* = 3 per group). Scale bar, 200 μm, 100 μm. (F) The mRNA expression levels of *MuRF1* and *Atrogin1* were quantified by qRT‐PCR (*n* = 3 per group). (G) The mRNA expression levels of *MyoD* and *MyoG* were quantified by qRT‐PCR (*n* = 3 per group). (H)The expression levels of MuRF1, Atrogin1, MyoD and MyoG were quantified by western blotting (*n* = 3 per group). (I) Relative expression of MuRF1 and Atrogin1 (*n* = 3 per group). (J) Relative expression of MyoD and MyoG. Relative expressions were normalized with GAPDH as internal control (*n* = 3 per group). The data were expressed as LSM ± SE. *P < 0.05; **P < 0.01; ***P < 0.001 by two‐tailed Student's *t*‐test.

To investigate the influence of OA on HFD‐induced obesity in C57BL/6 mice, we first measured a series of indicators including body weight, white adipose tissue (WAT), muscle, brown adipose tissue (BAT), and liver mass in HFD mice. Compared with CHOW mice, HFD mice had higher body weight, white adipose tissue (WAT) and BAT mass, but lower skeletal muscle mass (Fig. 1A,B). However, there was no significant difference in liver mass between CHOW mice and HFD mice (Fig. 1B). Further, glucose tolerance and insulin resistance tests were performed to investigate the effect of HFD on metabolism. The results showed that compared with CHOW mice, HFD mice exhibited impaired glucose tolerance and higher insulin resistance (Fig. 1C).

Next, we measured the cross‐sectional area (CSA) of perirenal white adipose tissue (pWAT) and TA muscle tissues by HE staining (Fig. 1D,E). The results showed that the average cross‐sectional area (CSA) of pWAT tissues increased and that of TA muscle tissues decreased in HFD mice, compared with CHOW mice. Further, we examined the mRNA and protein levels of factors associated with myogenesis and atrophy. Our data showed that the mRNA level of *MuRF1* (Fig. 1F) was significantly increased, but that of *MyoD* (Fig. 1G) was significantly decreased in HFD mice. However, there were no significant differences in the mRNA level of *Atrogin1* and *MyoG* between CHOW and HFD mice (Fig. 1F,G). The protein level of MuRF1 and Atrogin1 (Fig. 1H,I) was significantly increased in HFD mice, but that of MyoD and MyoG (Fig. 1H,J) was significantly decreased [31]. In summary, these results indicated that HFD resulted in obesity and skeletal muscle mass loss.

### OA treatment alleviates DEX‐induced muscle atrophy in C2C12 myoblasts

**Fig. 2:**
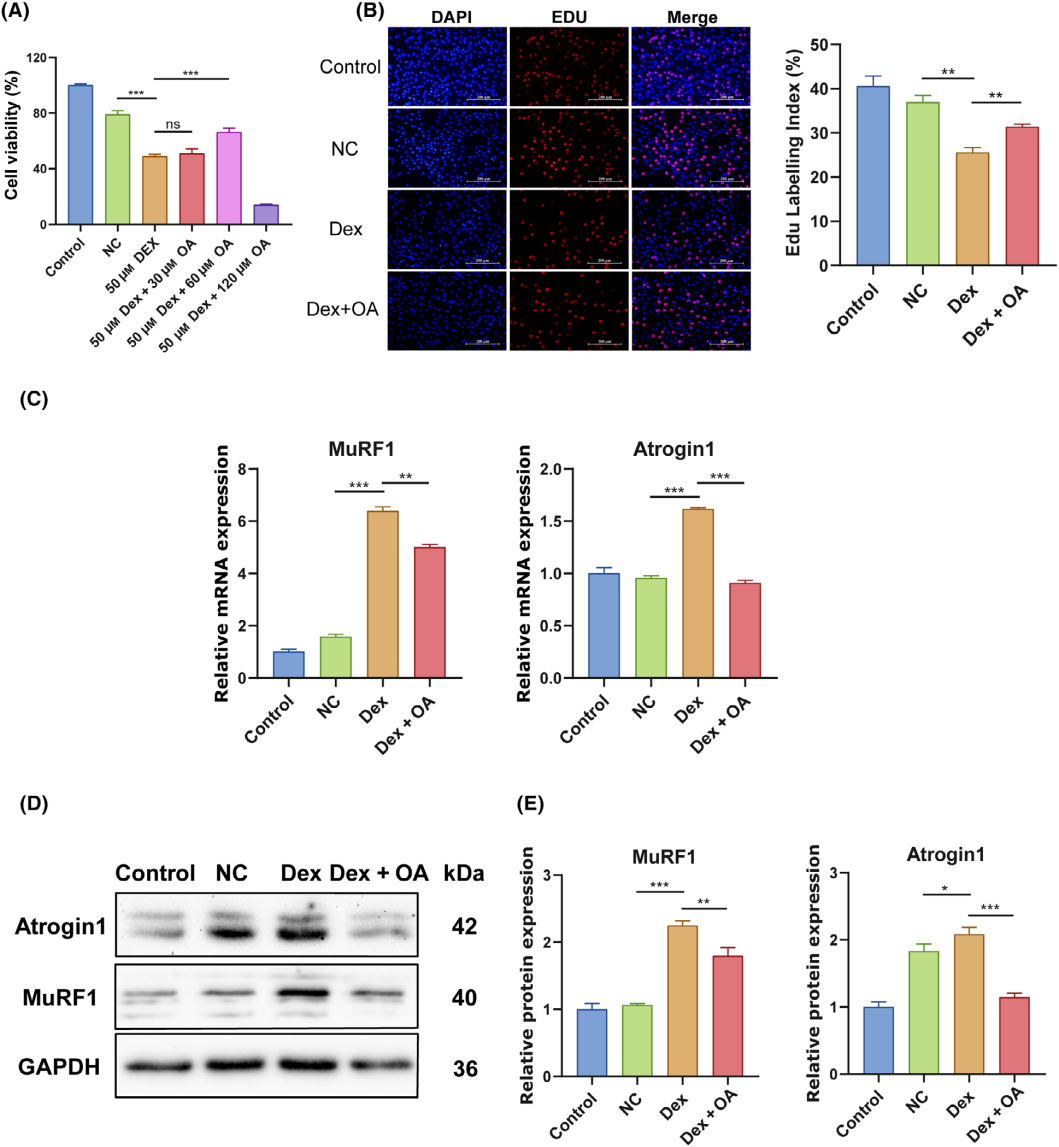
Effects of OA on Dexamethasone‐induced muscle atrophy in C2C12 myoblasts. (A) Effects of 50 μm Dex treatment and 50 μm Dex + 30, 60, 120 μm OA co‐treatment on C2C12 cells proliferation by CCK‐8 assay (*n* = 5 per group). (B) Effects of 50 μm Dex treatment and 50 μm Dex + 60 μm OA co‐treatment on C2C12 cells proliferation by EdU assay (*n* = 3 per group). Scale bar, 200 μm. (C, D) The mRNA expression (C) and protein expression (D) of MuRF1 and Atrogin1 in Control, NC, Dex, Dex + OA groups. Control group (without drug additions), NC group (anhydrous ethanol and dimethyl sulfoxide), Dex group (48 h treatment with 50 μm Dex), and Dex + OA group (12 h treatment with 50 μm DEX, followed by 36 h co‐treatment with 50 μm Dex and 60 μm OA) (*n* = 3 per group). (E) Relative expressions of MuRF1 and Atrogin1 were normalized with GAPDH as internal control (*n* = 3 per group). The data were expressed as LSM ± SE. *P < 0.05; **P < 0.01; ***P < 0.001 by two‐tailed Student's *t*‐test.

To investigate the influence of OA on muscle atrophy, we treated C2C12 myoblasts with 50 μm Dex to construct a muscle atrophy model. To determine the optimal concentration of OA for C2C12 cells treatment, we first treated C2C12 cells with 50 μm Dex for 12 h, and then co‐treated C2C12 cells with 30, 60 and 120 μm OA and 50 μm of Dex for 36 h. CCK‐8 assay results showed that 50 μm Dex + 60 μm OA co‐treatment significantly increased cell viability (Fig. 2A). EdU staining results showed that 50 μm Dex treatment significantly inhibited cell proliferation of C2C12 cells, and 50 μm Dex + 60 μm OA co‐treatment significantly promoted cell proliferation of C2C12 cells (Fig. 2B).

Next, we detected the expression of muscle‐degrading enzymes Atrogin1 and MuRF1 during DEX‐treated C2C12 myoblasts by qRT‐PCR and western blotting. The results showed that compared to the control cells, Dex‐treated cells exhibited a significant up‐regulation of the mRNA and protein expressions of MuRF1 and Atrogin1 (Fig. 2C,D). The 60 μm OA treatment significantly decreased the mRNA and protein expressions of MuRF1 and Atrogin1 in Dex‐stimulated C2C12 myoblasts (Fig. 2C–E). The above results indicated that OA treatment could alleviate Dex‐induced muscle atrophy in C2C12 myoblasts.

### OA treatment alleviates DEX‐induced muscle atrophy in C2C12 myoblasts through PI3K/Akt pathway

Further, we investigated signaling pathways related to protein synthesis, since protein synthesis is required for skeletal muscle development [32]. The PI3K/Akt pathway can not only regulate protein synthesis, but also be a key regulatory pathway for skeletal muscle growth. OA has been reported to regulate apoptosis and autophagy through the PI3K/Akt pathway [33, 34]. We hypothesized that OA might induce protein synthesis through PI3K/Akt pathway. Then, we investigated the expression of PI3K, p‐PI3K, Akt, and p‐Akt which were regulators of Atrogin1 and MuRF1, in the Dex‐treated C2C12 myoblasts by western blotting.

**Fig. 3:**
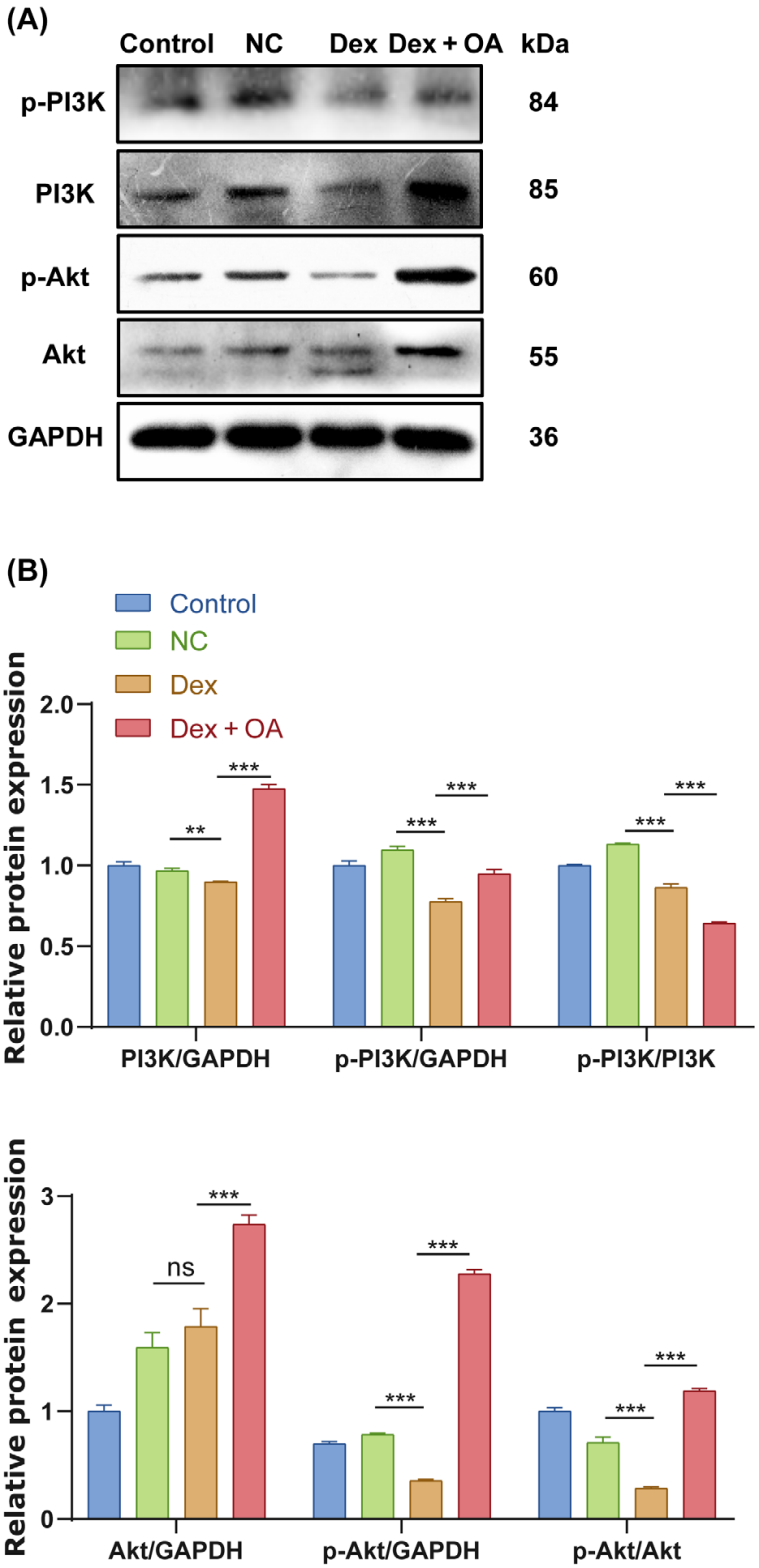
Regulatory effects of OA on PI3K/Akt signaling pathway in C2C12 myoblasts. (A) Expression of p‐PI3K, PI3K, p‐Akt, and Akt by western blotting (*n* = 3 per group). (B) Relative expression of PI3K/GAPDH, p‐PI3K/GAPDH, p‐PI3K/PI3K and Akt/GAPDH, p‐Akt/GAPDH, p‐Akt/Akt in C2C12 cells (*n* = 3 per group). The band density was analyzed by image j software. The data were expressed as LSM ± SE. **P < 0.01; ***P < 0.001 by two‐tailed Student's *t*‐test.

Interestingly, the expression of p‐PI3K/PI3K and p‐Akt/Akt was significantly lower in Dex‐stimulated C2C12 myoblasts than in control cells (Fig. 3A,B). After 60 μm OA treatment with Dex‐stimulated C2C12 cells, p‐PI3K/PI3K ratio did not increase (Fig. 3B). However, after 60 μm OA treatment with Dex‐stimulated C2C12 cells, the level of p‐Akt/Akt was significantly increased (Fig. 3B). These data suggested that PI3K/Akt signaling pathway played a role in OA‐mediated Dex‐induced C2C12 myoblasts.

### OA treatment reduces weight gain and increases skeletal muscle mass and size in HFD‐induced muscle atrophy mice

**Fig. 4:**
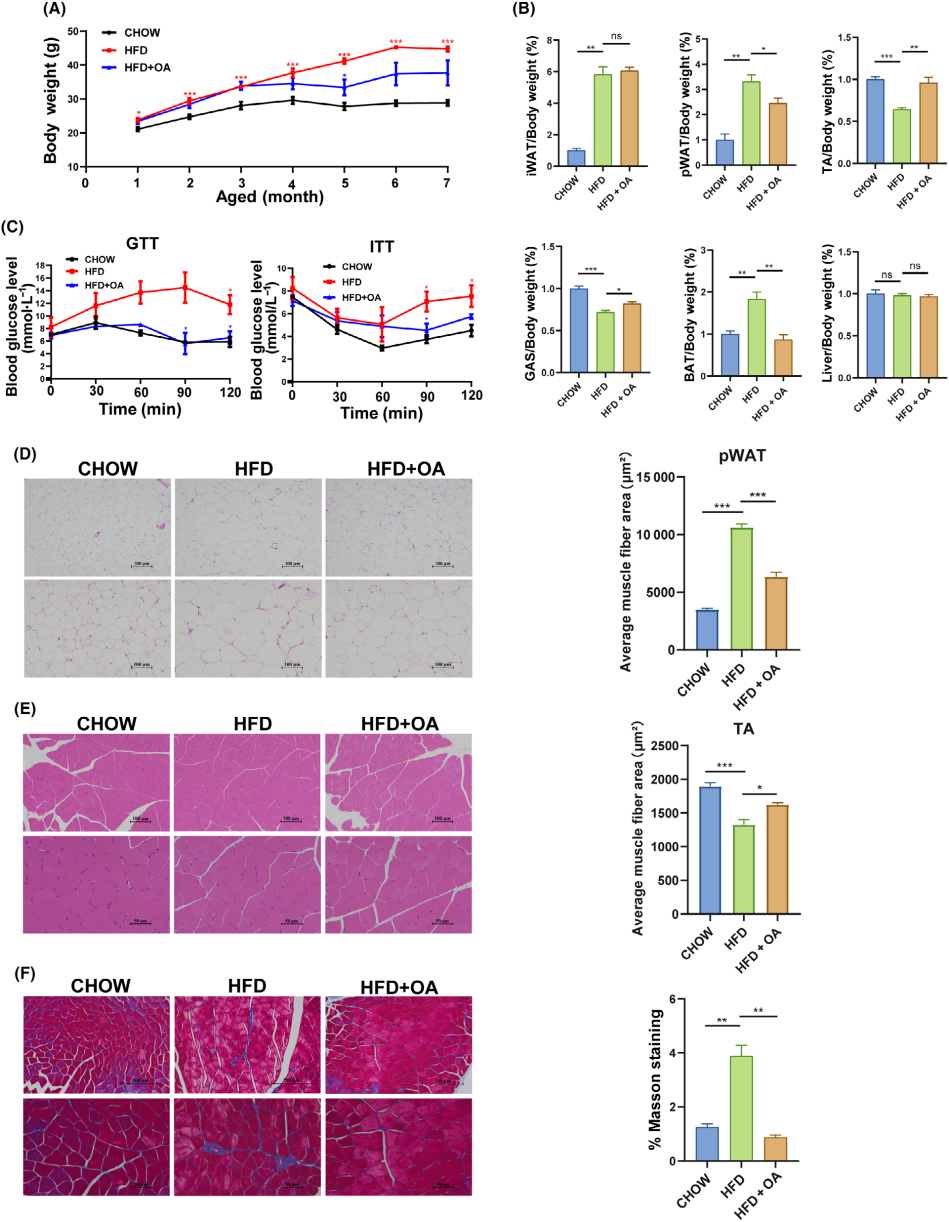
Effects of OA on body weight and tissue mass in HFD mice with muscle atrophy. (A) Comparison of body weight among CHOW, HFD, and HFD + OA groups (*n* = 4 per group). (B) Ratio of adipose, muscle, liver, and brown adipose tissue (BAT) to body weight in CHOW, HFD and HFD + OA groups (*n* = 4 per group). (C) Effects of OA on glucose tolerance and insulin resistance (*n* = 4 per group). (D) HE staining of perirenal white adipose tissue (pWAT). Comparison of average cross‐sectional area of pWAT among CHOW, HFD, and HFD + OA groups (*n* = 3 per group). Scale bar, 200 μm, 100 μm. (E) HE staining of Tibialis anterior (TA) muscle. Comparison of average cross‐sectional area of TA tissues among CHOW, HFD, and HFD + OA groups (*n* = 3 per group). Scale bar, 100 μm, 50 μm. (F) Masson staining of the tibialis anterior (TA) muscle (*n* = 3 per group). Scale bar, 200 μm, 50 μm. The data were expressed as LSM ± SE. *P < 0.05; **P < 0.01; ***P < 0.001 by two‐tailed Student's *t*‐test.

Based on the above‐mentioned *in vitro* experiment results, an *in vivo* experiment was performed to reveal the mechanism by which OA affected muscle atrophy. To investigate the effects of OA on muscle atrophy, we measured body weight, white adipose, skeletal muscle, brown adipose, and liver mass. The results showed that compared to CHOW group, HFD‐treated mice exhibited a significantly elevated body weight (Fig. 4A) but significantly decreased gastrocnemius (GAS) mass and TA muscle mass (Fig. 4B). The 50 mg·kg^−1^ OA treatment significantly increased the body weight at 5 months of age in mice and muscle mass of mice suffering from muscle atrophy. Meanwhile, the mass of white adipose tissue (WAT) and brown adipose tissue (BAT) in HFD group was significantly increased, while the mass of perirenal white adipose tissue (pWAT) and BAT in HFD + OA group was significantly decreased (Fig. 4B). However, there was no significant difference in inguinal white adipose tissue (iWAT) mass between HFD group and HFD + OA group. Meanwhile, no difference in liver tissue mass was observed among CHOW, HFD, and HFD + OA groups.

Compared with CHOW group, HFD group displayed lower glucose tolerance (Fig. 4C), and higher insulin resistance (Fig. 4C). However, 6‐month OA treatment significantly improved glucose intolerance and insulin resistance in HFD + OA group (Fig. 4C). These results showed that mice with muscle atrophy induced glucose intolerance and insulin resistance, and that OA treatment alleviated glucose intolerance and insulin resistance in mice with muscle atrophy.

Compared with the CHOW group, HFD group exhibited a significantly increased CSA of pWAT. Additionally, the OA treatment resulted in a lower CSA of pWAT in HFD + OA than in HFD group (Fig. 4D). CSA of TA muscle tissue was smaller in the HFD group than in the CHOW group. OA treatment enhanced TA muscle CSA, relative to the HFD group (Fig. 4E). As shown in Fig. 4F, the area of collagen fibrosis was significantly larger in HFD group than in CHOW group, and OA treatment significantly reduced the area of collagen fibrosis. It indicated that OA treatment increased the cross‐sectional area of TA tissues and decreased the area of collagen fibrosis in mice with muscle atrophy. The above results jointly suggested that HFD induced muscle atrophy in mice, that the OA treatment alleviated muscle atrophy, and that OA treatment improved glucose intolerance and insulin resistance in mice with muscle atrophy.

### OA treatment alleviates muscle atrophy in HFD‐induced obesity mice by regulating PI3K/Akt pathway

**Fig. 5:**
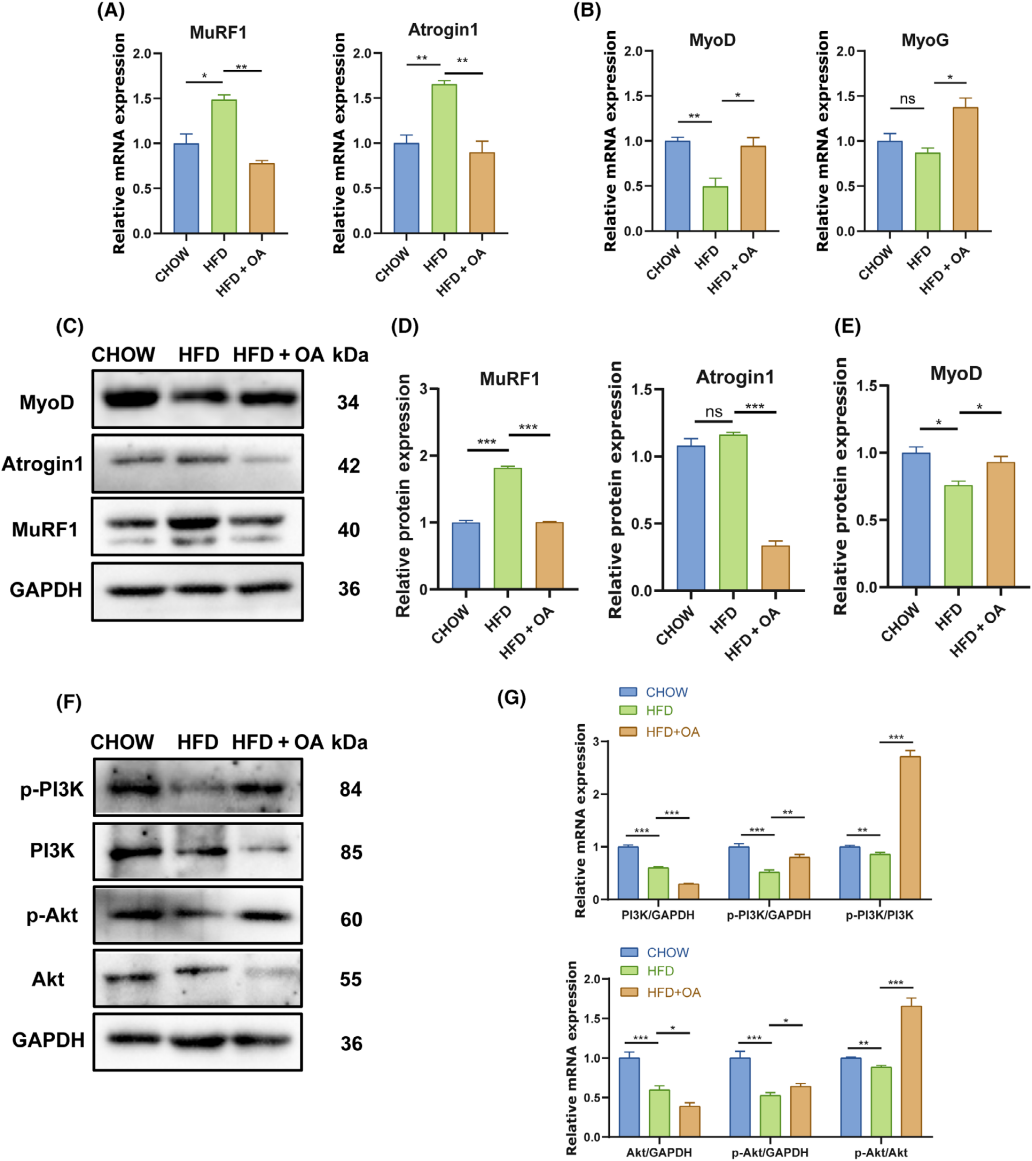
Effects of OA on muscle atrophy in HFD‐induced obese mice by regulating PI3K/Akt pathway. (A) Expression levels of *MuRF1* and *Atrogin1* quantified by qRT‐PCR (*n* = 3 per group). (B) Expression levels of *MyoD* and *MyoG* quantified by qRT‐PCR (*n* = 3 per group). (C) Expression levels of MyoD, MuRF1, and Atrogin1 quantified by western blotting (*n* = 3 per group). (D) Relative expression of MuRF1 and Atrogin1 with GAPDH as internal control (*n* = 3 per group). (E) Relative expression of MyoD with GAPDH as internal control (*n* = 3 per group). (F) Expression of p‐PI3K, PI3K, p‐Akt and Akt in C2C12 cells by western blotting (*n* = 3 per group). (G) Relative expression of PI3K/GAPDH, p‐PI3K/GAPDH, p‐PI3K/PI3K and Akt/GAPDH, p‐Akt/GAPDH, p‐Akt/Akt (*n* = 3 per group). The data were expressed as LSM ± SE. *P < 0.05; **P < 0.01; ***P < 0.001 by two‐tailed Student's *t*‐test.

To further verify the above results that OA treatment alleviated muscle atrophy, we conducted qRT‐PCR and western blotting assay. The results revealed that the HFD significantly up‐regulated the mRNA expressions of *MuRF1* and *Atrogin1*, but OA treatment reversed their up‐regulation (Fig. 5A). The protein levels of MuRF1 were consistent with mRNA levels (Fig. 5C,D). Moreover, the protein expression of Atrogin1 was not significantly different between CHOW group and HFD group, but it was significantly reduced after OA treatment (Fig. 5C,D). The mRNA and protein expressions of MyoD in TA tissues of HFD group were significantly lower than those of CHOW group (Fig. 5B,C,E). However, OA treatment significantly increased the mRNA and protein expression levels of MyoD in HFD + OA group, compared with HFD group (Fig. 5B,C,E). The qRT‐PCR results showed that there was no difference in *MyoG* mRNA expression between CHOW group and HFD group, and the mRNA expression level of *MyoG* was significantly increased after OA treatment (Fig. 5B). Based on the above results, it could be concluded that OA treatment reduced the expression of factors related to skeletal muscle atrophy and increased the expression of factors related to skeletal muscle development in mice with muscle atrophy.

To investigate whether OA affected skeletal muscle development through PI3K/Akt pathway, we analyzed the expressions of PI3K, Akt and p‐PI3K, p‐Akt proteins in mice suffering from muscle atrophy. We found that the expressions of p‐PI3K/PI3K and p‐Akt/Akt was significantly declined in the TA tissues of mice with muscle atrophy (Fig. 5F,G). However, OA treatment significantly increased p‐PI3K/PI3K and p‐Akt/Akt ratio (Fig. 5G). These results suggested that OA promoted protein synthesis potentially by regulating the PI3K/Akt signaling pathways in atrophied muscle, which might explain why OA alleviated muscular atrophy in HFD‐induced obesity mice.

## Discussion

Obesity has been reported to be related to dysfunctions of skeletal muscle, and it can also lead to decreased muscle mass and physiological dysfunctions [35, 36]. It is well‐known that HFD induces obesity, and in turn obesity induces muscle atrophy in animals [37]. Although muscle atrophy has a wide clinical impact, and much effort has been made to explore the drugs against muscle atrophy, no effective medications are available.

Recent evidence has depicted potential applications of OA to obesity and other metabolic diseases through the regulation of pro‐inflammatory cytokines [21, 22, 23, 24]. In line with previous reports, our experimental data showed that HFD mice exhibited obesity, muscle atrophy, an increased body weight and white adipose tissue mass, but a decreased muscle mass and muscle CSA [38, 39]. Additionally, we also observed that obesity induced glucose intolerance and insulin resistance, which was consistent with previous findings [40]. However, we found that OA treatment could improve glucose intolerance and insulin resistance in mice with muscle atrophy. At the molecular level, the mRNA expression of *MuRF1* and *Atrogin1* increased in HFD mice, which was consistent with previous studies [41]. In conclusion, OA improved muscle atrophy by increasing the protein expression of MyoD and decreasing the protein expression of MuRF1 and Atrogin1.

It is crucial for maintaining muscle mass and functions to keep the balance between protein synthesis and protein degradation in skeletal muscle [42]. When this balance is lost, obesity, muscle atrophy, and other metabolic diseases generally occur [43]. Muscle protein degradation is mainly mediated by two pathways, namely, the ubiquitin proteasome and the autophagy lysosomal system [44, 45]. In this study, we mainly investigated the protein degradation pathway of ubiquitinated protease to reveal the protective effects of OA on muscle atrophy [46, 47].

Mechanically, the activation of PI3K/Akt signaling pathway can improve muscle development retardation, while the inhibition of Akt activity with drugs will promote skeletal muscle protein hydrolysis and muscle atrophy [48]. In addition, the PI3K/Akt pathway is also involved in protein synthesis and degradation. What causes skeletal muscle development retardation includes not only Akt pathway, but also NF‐κB, AMPK, FOXO, STAT3, and other signaling pathways [49, 50, 51, 52, 53]. In this study, we found that PI3K/Akt signaling pathway played a role in the process of skeletal muscle atrophy, which was consistent with previous reports [54].

Our *in vivo* and *in vitro* experiments indicated that OA inhibited the expression of muscle atrophy‐related proteins and enhanced the expression of myogenic development‐related proteins. We further demonstrated that OA increased protein synthesis through the PI3K/Akt signaling pathway, ultimately alleviating muscle atrophy. In the HFD + OA group, the total protein levels of PI3K and Akt in TA muscle decreased. It indicated that OA decreased the total protein levels of PI3K and Akt in mice with muscle atrophy. These results are inconsistent with OA‐treated DEX‐induced muscle atrophy in C2C12 myoblasts. This may be due to the fact that the animal organism is a complex environment, and the total proteins levels of PI3k and Akt are affected by a variety of proteins and other stimuli. Compared with the CHOW group, the HFD group decreased the levels of p‐PI3K/PI3K and p‐Akt/Akt. Conversely, OA treatment increased the expression of p‐PI3K/PI3K and p‐Akt/Akt, thus promoting protein synthesis, eventually alleviating muscle atrophy. In *in vitro* experiments, we constructed a Dex‐induced muscle atrophy in C2C12 cells. Consistent with the results of *in vivo* experiments, the Dex treatment inhibited the PI3K/Akt signaling pathway. The qRT‐PCR and western blotting results showed that the expression of muscle atrophy‐related MuRF1 and Atrogin1 was inhibited, thus alleviating DEX‐induced muscle atrophy in C2C12 cells. After OA treatment, the p‐PI3K/PI3K ratio was lower than that in the DEX group. This result does not evidence of activation of PI3K by OA. However, the p‐Akt/Akt ratio was greater in the DEX + OA group than in the DEX group. The results showed that OA increased the activation of Akt. This may be due to the activated of Akt protein by other related proteins, such as: (insulin‐like growth factor 1) IGF‐1, FoXo, PIP3 [32, 55]. These results suggested that OA alleviated muscle atrophy by regulating the PI3K/Akt pathway.

In conclusion, this study only focused on PI3K/Akt signaling pathway through which OA alleviated muscle atrophy. The future studies are suggested to investigate more related pathways. Specific component alleviating muscle atrophy in OA compound remains to be further explored. In the present study, we demonstrated that OA attenuated muscle atrophy by regulating PI3K/Akt signaling. OA increased skeletal muscle mass and size by increasing muscle protein synthesis‐related genes and suppressing muscle protein degradation‐related genes. Our findings provide theoretical basis for prevention and treatment of muscle atrophy by OA.

## Conflict of interest

The authors declare no conflict of interest.

### Peer review

The peer review history for this article is available at https://www.webofscience.com/api/gateway/wos/peer‐review/10.1002/2211‐5463.13780.

## Author contributions

Conceptualization, HW, YY; Data curation, YS, XW; Formal analysis, TZ; Funding acquisition, HW, YY; Methodology, YS, HS and YW; Project administration, HW; Resources, HZ, ZY; Software, XH, MZ and TM; Validation, TF, JL and XL; Writing – original draft, YS and YY.

## Supporting information


**Table S1.** Primer sequences.

## Data Availability

All the data generated or analyzed during this study are included in the published article.
