# Supplementary material for: Oleanolic acid alleviates obesity‐induced skeletal muscle atrophy via the PI3K/Akt signaling pathway
Source: FEBS Open Bio. 2024 Feb 17;14(4):584–97. doi: 10.1002/2211-5463.13780 (PMC10988678; doi:10.1002/2211-5463.13780)
Supplement: Supplementary file 1 — Table S1. Primer sequences. [file FEB4-14-584-s001.docx]

| Gene | Primer sequence |
| --- | --- |
| 36B4 | Forward:TGGAGACAAGGTGGGAGCC |
|  | Reverse:CACAGACAATGCCAGGACGC |
| MuRF1 | Forward:TGATTCCTGATGGAAACGCT |
|  | Reverse:TCATTGGTGTTCTTCTTTACCCTC |
| Atrogin1 | Forward:TAGCATCGGTATGACTAAGT |
|  | Reverse:AGTCATATGGCAAGCATAC |
| MyoD | Forward: GAATGGCTACGACACCGCCTACTAC |
|  | Reverse: ACGGGGTCTGGGTTCCCTGTT |
| MyoG | Forward: AACTACCTTCCTGTCCACCTTC |
|  | Reverse:CACAGACTTCCTCTTACACACCT |

Table S1
